# Self-management and its associated factors among people living with diabetes in Blantyre, Malawi: a cross-sectional study

**DOI:** 10.12688/aasopenres.12992.1

**Published:** 2019-09-19

**Authors:** Chimwemwe Kwanjo Banda, Belinda T. Gombachika, Moffat J. Nyirenda, Adamson Sinjani Muula

**Affiliations:** 1 School of Public Health and Family Medicine, University of Malawi College of Medicine, Blantyre, Malawi; 2 Medical and Surgical Nursing, University of Malawi, Kamuzu College of Nursing, Blantyre, Malawi; 3 Uganda MRC/UVRI Research Unit, Entebbe, Uganda; 4 NCD-Brite Consortium, University of Malawi College of Medicine, Blantyre, Malawi; 5 Africa Center of Excellence in Public Health and Herbal Medicine, University of Malawi College of Medicine, Blantyre, Malawi

**Keywords:** diabetes education, glycemic control, physical activity, healthy diet, foot care, social cognitive theory, environmental factors, social support

## Abstract

**Background:** Self-management is key to the control of glycaemia and prevention of complications in people living with diabetes. Many people living with diabetes in Malawi have poorly controlled glucose and they experience diabetes-related complications. This study aimed to assess diabetes self-management behaviours and to identify factors associated with it among people living with diabetes at Queen Elizabeth Central Hospital (QECH), Blantyre, Malawi.

**Methods:** This cross-sectional study recruited 510 adults attending a diabetes clinic at a teaching referral hospital in southern Malawi. The social cognitive theory was applied to identify factors associated with following all recommended self-management behaviours. Data on participants’ demographics, clinical history, diabetes knowledge, self-efficacy, outcome expectations, social support, environmental barriers and diabetes self-management were collected. Univariate and multivariate logistic regression analyses were conducted to identify factors associated with following all self-management behaviours.

**Results:** The mean age of participants was 53.6 (SD 13.3) years. Self-reported medication adherence within the last seven days was 88.6% (n=494); 77% reported being physically active for at least 30 minutes on more than three days in the previous seven days; 69% reported checking their feet every day and inspecting inside their shoes; 58% reported following a healthy diet regularly. Overall, only 33% reported following all the self-management behaviours regularly. Multiple logistic regression analysis showed that self-efficacy was the only social cognitive factor associated with following all the self-management practices (p < 0.001).

**Conclusions:** Participants in our study were not consistently achieving all self-management practices with dietary practices being the least adhered to behaviour by many. To improve self-management practices of people living with diabetes, current health education programs should not only aim at improving diabetes related knowledge but also self-efficacy. Adopting interventions that promote self-efficacy in diabetes patients such as exposure to role models, peer education, providing positive feedback, and counselling is recommended.

## Introduction

Diabetes mellitus significantly contributes to morbidity and mortality from non-communicable diseases in Malawi ^1^. Diabetes is the ninth-leading cause for admissions in adult medical wards at Queen Elizabeth Central Hospital (QECH) in Blantyre, the largest public teaching hospital in Malawi ^2^. The inpatient mortality for the people admitted due to diabetes at QECH is about 19% ^2^. A previous survey at QECH by Cohen *et al.*, conducted 10 years before the present study found that 74% of people living with diabetes had poorly controlled sugar levels and many suffered from diabetes related complications ^3^. This previous survey also found that 45% of patients living with diabetes had poor dietary practices. Among the patients that were on insulin, about 22% had problems with proper injection technique ^3^. However, Cohen *et al.* did not report on other self-management practices such as medication adherence, self-monitoring of blood glucose, foot care, smoking habits and exercises. Subsequent to the study by Cohen *et al.*, clinical guidelines and protocols for the management of diabetes and nurse-led education classes for diabetes patients were introduced at QECH ^3^. The nurse-led education classes offer lessons to people living with diabetes on lifestyle practices related to diet, exercises, medication adherence, smoking cessation, foot care, and management of symptoms generated by the disease to help keep diabetes under control and to prevent its complications.

Although the study by Cohen *et al.* had shown that people living with diabetes in Malawi had poorly controlled glucose ^3^, little was known about their self-management behaviours especially regarding diet, exercising, self-monitoring of blood glucose, medication adherence, and foot care. Furthermore, since the introduction of the nurse-led diabetes education classes, no follow-up study was conducted to evaluate if there has been an impact on people’s diabetes self-management behaviours.

The conceptual framework guiding the study was adapted from social cognitive theory (SCT) by Albert Bandura ^4^. Recent studies continue to show that the propositions made in the SCT on determinants of health behaviour remain valid to date, not only for diabetes but many other chronic conditions. A systematic review of randomized controlled trials of SCT-based interventions showed effectiveness in improving diet and exercise behaviour in cancer survivors ^5^. The social cognitive theory outlines several factors that are key to the acquisition of knowledge and skills which influence health and wellbeing of individuals. Some key concepts of the social cognitive theory are self-efficacy, health knowledge, health goals, outcome expectations, and environmental impediments and facilitators ^4, 6^. These key concepts are the factors that influence human action, motivation and wellbeing and hence are hypothesized to be associated with diabetes self-management for this present study ^6^. The aim for this study was to assess the level of self-management and identify factors associated with practicing all self-management behaviours among adults living with diabetes at QECH. This is part of a larger study exploring self-management practices and experiences among people living with diabetes attending the QECH diabetes clinic.

## Methods

A cross-sectional design was used to collect demographics, clinical, diabetes knowledge, self-efficacy, outcome expectations, social support, environmental barriers and diabetes self-management data. Ethical approval for the study was granted by the College of Medicine Research and Ethics Committee (Ref: P.08/17/229). All participants provided written informed consent to participate in the study.

### Inclusion and exclusion criteria

Recruitment of clients was done on diabetes clinic days at QECH. The diabetes clinic at QECH runs once a week. Clients were eligible to participate if they were aged 18 years and above, had clinically confirmed type 1 or type 2 diabetes mellitus for over six months and were available at the clinic between 9am and 1pm, the time when data were being collected. Clients were excluded if they had cognitive impairment or communication difficulties, had lived with diabetes for less than 6 months or were acutely ill. To avoid selection bias, systematic sampling was used and an invitation was made to every third person on the queue who met the recruitment criteria until the required sample size was met.

### Sample size considerations

It was assumed that the proportion of participants achieving good self-management could be 0.26 (26%), based on the previous study at QECH by Cohen *et al.* (2010) ^3^. The proportion was estimated to be within 0.04 (4%), using the 95% confidence level a sample of 462 patients living with diabetes would be required. The final sample size of 510 was obtained after a 10% adjustment for refusals and to account for potential confounding factors (age, sex, type of diabetes and duration of diabetes since diagnosis).

### Data collection and instruments

Data were collected between November 2017 and May 2018 using an interviewer-administered questionnaire ^7^. The questionnaire was administered by the first author and five other trained research assistant all of whom have a background in nursing. To mitigate social desirability bias whereby respondents tend to over-report healthy behaviours or under-report unhealthy behaviours, during recruitment the participants were informed of the anonymity of the data and how their participation or refusal to participate in the study would not affect their care at the diabetes clinic ^8^. Furthermore, the researcher and research assistants did not wear nurses’ uniform during data collection to create a neutral environment. The questionnaire collected data on the participants’ demographics, clinical history, social cognitive theory constructs (diabetes knowledge, self-efficacy, outcome-expectations, environmental barriers to proper self-management and social support) and self-management and it is available as *Extended data* on Figshare ^7^. Clinical data were extracted from participants’ health passport books and included weight, height, body mass index (BMI), blood pressure reading for that day, fasting blood glucose (FBG) reading for that day and for the client’s last two clinic visits, creatinine checked within the last 12 months, time since diabetes diagnosis, type of diabetes, type of treatment, diabetes complications, and if there were any comorbidities including HIV status.

To measure self-management, we adapted ten items from the Summary of Diabetes Self-Care Activities (SDSCA) measure and one item from the expanded version of the SDSCA developed by Toolbert *et al.* ^9^. We used all four items of the SDSCA on diet. We dropped one of the two items on exercises as the participants who took part in the content validation of the questionnaire felt that the items were asking the same thing. The items on blood sugar testing were dropped as content experts who reviewed the tool felt that the questions were not applicable in the Malawian setting since many people may not have a personal glucometer. Instead we included a question that asked the participants if they have a glucometer at home. Reliability and validity of the SDSCA measure has been proved from previous studies with a high correlation with other scales of self-care ^9^. The adapted SDSCA assessed level of self-care related to diet (four questions), exercise (one question), blood sugar testing (one question), foot care (two questions), smoking (two questions) and medication (one question). For each subscale, the respondent was asked to mention number of days they performed a particular activity in the past one week. Reverse scoring was done for the question on fat intake. For all the questions, a higher number of days indicated satisfactory self-management.

Diabetes knowledge was measured using items adapted from the Diabetes Knowledge Questionnaire (DKQ) ^10^. We used all the 24 items of the DKQ to measure knowledge on causes, signs and symptoms, pathophysiology and treatment of diabetes. The tool had showed construct validity and reliability in a Mexican-American population with Cronbach’s coefficient α of 0.78 ^10^. For each item, the respondents answered either “yes”, “no” or “I don’t know”. The total number of correct answers was calculated at the end to obtain the knowledge score.

Self-efficacy is a person’s belief or judgement in their ability to accomplish specific acts ^11^. The Self-efficacy for Diabetes Tool ^12^ was used to measure self-efficacy. All the eight items of the tool were used to asks of the respondents’ confidence to perform various diabetes self-management tasks on a scale of 1 to 10, where 1 was “not at all confident” and 10 was “totally confident” ^13^. Scoring of the scale was based on the mean of at least six items with higher scores indicating higher self-efficacy. This tool had been used in previous studies with a Cronbach’s coefficient α of 0.85 and a test-retest validity of 0.8 ^13^.

Outcome expectations refers to a persons’ belief or anticipated result for doing a particular behaviour ^6^. The Outcome expectations were assessed using items adapted from the multidimensional diabetes questionnaire ^14^. We adapted all six items related to outcome expectations from the questionnaire to ask participants’ perceptions on the effects of performing particular self-care activities on their glucose control or prevention of diabetes related complications. The scores ranged from 1 (not at all important) to 10 (totally important). Scoring of the scale was based on the mean of the six items with higher scores showing more positive expectancies. A previous study assessing the validity of this test found a Cronbach’s coefficient α of 0.86 ^14^.

Environmental factors that were assessed were social support and barriers to self-management. Social support is a multidimensional construct that refers to “a network of family, friends, neighbours, and community members that is available in times of need to give psychological, physical, and financial help” ^15^. Social support was assessed using a nine-item measure of social support with a five-point-Likert scale. The tool assessed availability of emotional, informational support, networking support and sources of social support ^16^. This tool was adapted from the Medical Outcomes Study social support and the items showed reliability (Cronbach’s coefficient α ranging 0.74 to 0. 93) and construct validity ^17^. Scoring was based on the frequencies of each item.

We used 24 items out of the 31 items of the environmental barriers to diabetes adherence tool ^18^ to assess environmental barriers of the participants. Six items on self-glucose monitoring were dropped during content validation of the tool to suit context as most people living with diabetes in Malawi do not have personal glucometers. One other item that stated “I feel sore and stiff” was dropped as participants in the content validation exercise of the Chichewa version felt that the item had the same meaning with the item that stated “I don’t feel well”.

### Data analysis

Data were entered into a Microsoft Access database then exported into Stata version 14.0 for cleaning and analysis. Descriptive statistics were used to show proportions and 95% confidence interval (CI) for categorical factors and mean and standard deviations (SD) for continuous factors that were normally distributed. Median and interquartile range (IQR) were calculated for factors that were not normally distributed. The outcome variable was self-management as measured by the SDSCA. Participants were categorized as having adequate self-management behaviour if they were adherent to all four self-management practices (diet, exercise, medication and foot care). There were only 14 people who were not on any diabetes medication, who were therefore excluded in the final analysis. Adherence to blood glucose self-monitoring was not assessed as only 12% of the participants had personal glucometers and all reported not to have measured themselves in the last seven days. Smoking status was not included as there were only three active smokers. Univariate logistic regression was done to investigate associations between demographics, clinical and social cognitive factors with the outcome variable. Chi-square (or Fisher’s exact) test and t-test (Wilcoxon rank sum test) were used for testing association between the binary outcome of self-management behaviour with categorical explanatory factors and continuous factors, respectively. Factors that showed association with adequate self-management at alpha 0.1 or less were included in the multivariate logistic regression model. Participants’ sex, diabetes type and duration of diabetes diagnosis were included in the multivariate logistic regression model because they were believed to be possible confounders. Associations were considered significant at alpha less than 0.05.

## Results

### Participant background

A total of 554 clients were selected and invited to participate in the study using systematic random sampling, of which 538 met the recruitment criteria and 28 refused to participate. In total, 510 consented to participate, representing a response rate of 95%. Overall, there were more females (82%). A total of 14 participants were excluded from the final analysis for having no data on medication adherence. Table 1 contains the demographic characteristics and in Table 2 are the clinical characteristics of the study participants.

**Table 1.** Participants demographic characteristics at QECH, Malawi.

Demographic Characteristics	Characteristic	n *	%
Sex	Male	91	17.84
	Female	419	82.16
Age, years	Mean (SD)	53.65 (13.31)	
Marital status	Never married	14	2.75
	Married	220	64.71
	Divorced	39	7.65
	Widowed	119	23.33
	Separated	8	1.57
Education	Never been to school	54	10.59
	Primary school	249	48.82
	Secondary school	180	35.29
	Tertiary education	27	5.29
Occupation	Employed	51	10
	Farming	64	12.55
	Small scale business	214	41.96
	Unemployed	148	29
	Retired	33	6.47

*Unless indicated. SD, standard deviation.

**Table 2.** Clinical characteristic of participants at QECH, Malawi.

Variable Characteristics	Characteristic	n *	%
Duration	5 years or less	272	54.08
	6 – 10 year	130	25.84
	11 – 15 years	57	11.33
	More than 15 years	44	8.75
Diabetes type	Type 1	56	10.98
	Type 2	443	86.86
	Unknown	11	2.16
Treatment	Insulin only	92	18.07
	Oral agents	348	68.37
	Insulin and oral agents	55	10.81
	Diet and exercise only	14	2.75
Complications	None	208	40.78
	One	229	44.9
	Two or more	73	14.31
HIV status	Negative	406	79.61
	Positive	76	14.90
	Unknown	28	5.49
Comorbidities	None	181	35.49
	One	306	60
	Two or more	23	4.51
BMI	Underweight	26	5.27
	Normal	152	30.83
	Overweight	162	32.86
	Obese	153	31.03
FBG	Median (IQR)	171.37 (129.24–234.51)	
Systolic BP	Median (IQR)	131 (118–146)	
Diastolic BP	Median (IQR)	84 (75–91)	

*Unless indicated. BMI, body mass index; BP: blood pressure; FBG: fasting blood glucose; IQR, interquartile range.

### Participant questionnaire responses

The median knowledge score on the diabetes knowledge questionnaire was 14 (IQR 12–16) with lowest knowledge scores being on causes of diabetes, importance of diet and exercising and recognition of hypoglycemia or hyperglycemia. The median self-efficacy score was 8.6 (IQR 7.5–9.5), and the participants had lower self-efficacy on eating evenly spaced meals regularly and exercising for at least 30 minutes three times a week. The median for outcome expectations score was 10 (IQR 10–10), suggesting that participants had positive expectations in following recommended self-management behaviours. The median social support score was 4.9 (IQR 2.9–5). The most commonly mentioned sources of social support were spouses and daughters. The median score for environmental barriers to self-care was 1.5 (IQR 1.3–1.8). Barriers to medication were infrequent with only 7% of the participants reporting encountering barriers at any point. Barriers to healthy diet and exercising were reported by 37% and 33% of the participants respectively.

**Figure 1.**
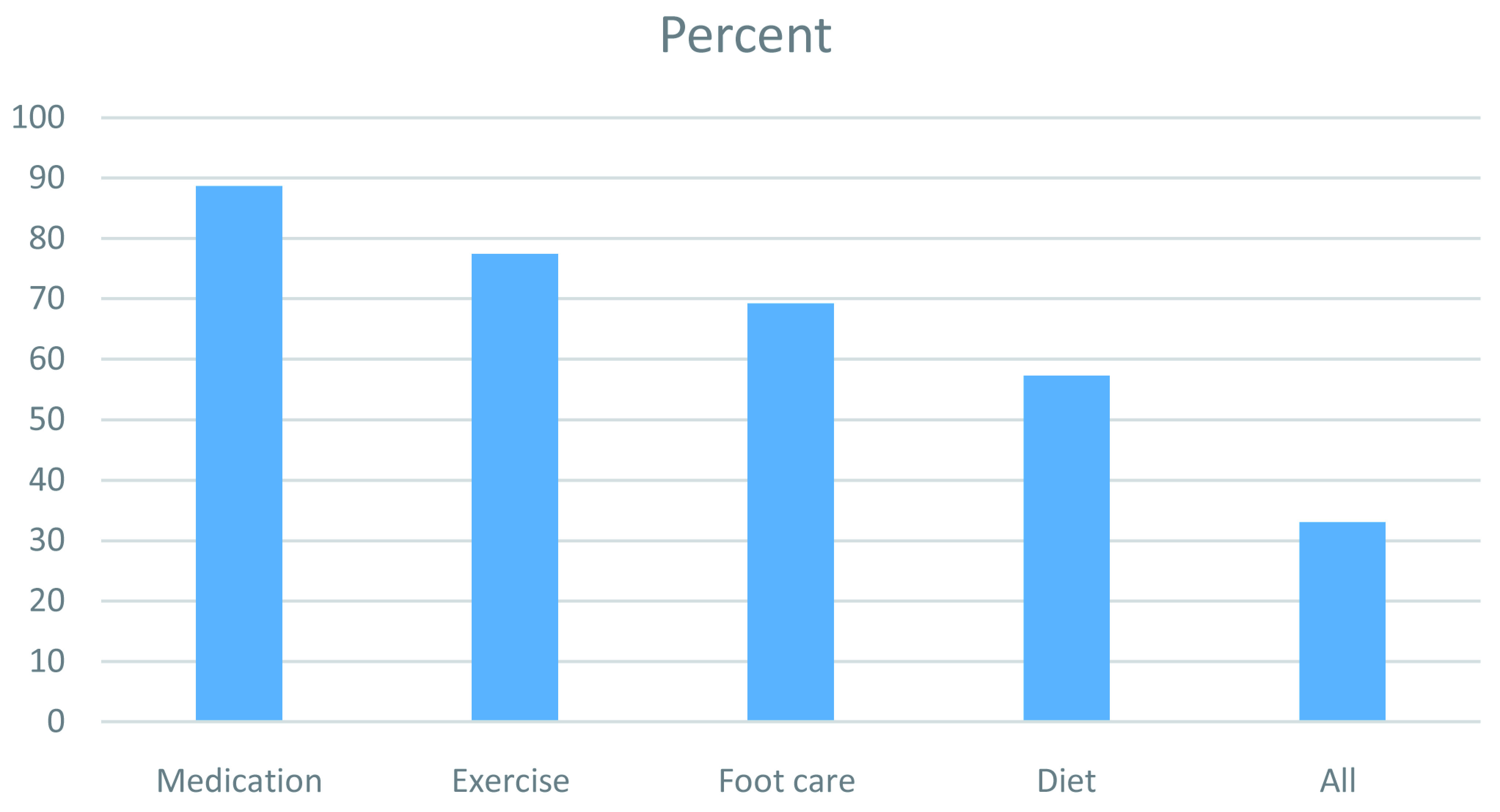
Percentage of participants reporting adherence to self-management behaviours.

Most participants reported taking their medication everyday as recommended (88.66%) and also reported being physically active for at least 30 minutes on three or more days per week (71.41%). Physical activities reported included walking or engaging in one’s daily duties. Daily foot care was reported by 69.23% of the participants. For the general diet, 57.28% of the participants reported a healthy diet 6–7 days per week. On the specific diet, none of the participants reported taking at least five portions of fruits and vegetables per day, while 48.53% reported not to have taken any high fat food on any day in the previous seven days. Participants were considered to have satisfactory self-management behaviour if they reported regular adherence to the general diet, exercising, foot care and medication intake. Only 33% of the participants were adherent to all the four self-management behaviours. Figure 1 shows the percentage of participants who reported adherence to specific self-management behaviours and all self-management behaviours. All responses are given as *Underlying data* ^19^.

### Univariate and multivariate analysis

To investigate the factors associated with adhering to all the self-management behaviours, univariate and multivariate regression analyses were done. The unadjusted logistic regression analyses showed that satisfactory self-management was associated with self-efficacy, social support, outcome expectations and diabetes barrier score (the results are shown in Table 3).

**Table 3.** Associations between demographic, clinical and social cognitive theory constructs with satisfactory self-management.

Variable	Characteristic	Odds ratio	95% CI	p-value
Sex	Female	1.007	0.613–1.655	0.979
Age (in years)	Every additional year	1.000	0.986–1.014	
Marital status	Never married	Reference		
	Married	3.100	0.682–14.103	0.143
	Divorced	3.36	0.656–17.212	0.146
	Widowed	2.530	0.538–11.900	0.24
	Separated	10	1.260–79.339	0.029
Education	Never been to school	Reference		
	Primary school	1.728	0.861–3.470	0.124
	Secondary school	1.892	0.925–3.869	0.081
	Tertiary education	0.877	0.270–2.848	0.827
Occupation	Employed	Reference		
	Farming	0.555	0.253–1.216	0.141
	Small scale business	0.707	0.279–1.794	0.466
	Unemployed	0.539	0.283–1.024	0.059
	Retired	0.705	0.363–1.371	0.303
BMI	Underweight	0.822	0.305– 2.210	0.697
	Normal	Reference		
	Overweight	1.367	0.844–2.213	0.204
	Obese	1.283	0.786–2.094	0.319
Systolic BP	every additional unit	1.002	0.994–1.011	0.556
Diastolic BP	every additional unit	0.999	0.985– 1.014	0.932
FBG	every additional unit	0.999	0.999– 1.001	0.271
Creatinine	Every additional unit	1.362	0.895– 2.073	0.149
Duration	5 years or less	Reference		
	6–10 year	1.034	0.657–1.627	0.886
	11–15 years	1.387	0.764–2.515	0.282
	More than 15 years	1.0344	0.520–2.059	0.923
Diabetes type	Type 1	Reference		
	Type 2	0.796	0.447–1.418	0.439
	Unknown	1.111	0.280– 4.399	0.881
Diabetes treatment	Insulin only	Reference		
	Oral agents	0.925	0.571–1.501	0.754
	Insulin and oral agents	0.839	0.410– 1.715	0.63
	Diet and exercise only	omitted		
Complications	None	Reference		
	One	0.968	0.648–1.447	0.875
	Two or more	0.694	0.380–1.265	0.233
Comorbidities	None	Reference		
	One	0.969	0.654–1.437	0.876
	Two or more	0.285	0.081–0.998	0.05
HIV status	Negative	Reference		
	Positive	1.303	0.783–2.167	0.309
	Unknown	1	0.440–2.273	1
Knowledge	Every additional score	0.960	0.894–1.030	0.416
Self-efficacy	Every additional score	1.499	1.288–1.744	0.0001
Outcome expectations	Every additional score	1.386	0.954– 2.013	0.087
Social support	Every additional score	1.276	1.031– 1.579	0.025
Total barrier score	Every additional score	0.536	0.321–0.893	0.017

CI, confidence interval; BMI, body mass index; BP, blood pressure; FBG, fasting blood glucose.

In the multivariate logistic regression model, we adjusted for age, sex, duration since diabetes diagnosis and type of diabetes. The results showed that self-efficacy was the only significant factor associated with satisfactory self-management (p < 0.001). For a one-unit increase in the self-efficacy score, the odds of having satisfactory self-management increase by 1.5 (CI 1.2 – 1.7).

## Discussion

This study applied the social cognitive theory to assess self-management behaviours and its associated factors among patients living with diabetes at an urban diabetes clinic in Blantyre, Malawi. Medication adherence was highest of all the self-management behaviours that were assessed. High rate of adherence to medication have also been reported in previous studies from the USA ^9^, Ethiopia ^20, 21^ and rural Malawi ^22^. Our results suggest that people living with diabetes attending the QECH diabetes clinic have fewer environmental barriers to medication adherence than to other self-care practices. The high levels of adherence to medication could also suggest that people living with diabetes prioritize medication intake over other self-management behaviours. Although medication adherence is associated with better glycemic control ^22^, it should be accompanied with lifestyle modifications for better results ^23, 24^.

A total of 71% of the participants also reported being physically active for at least 30 minutes three times a week as part of their daily work. The level of physical activity among the participants in our study was lower compared to findings from a population-based survey that was conducted in Malawi which reported that 90.5% adults were physically active ^25^. Engaging in regular physical activity among people living with diabetes contributes to cardiorespiratory fitness, improved glycaemic control, decreased insulin resistance, improved blood lipid profile and improved blood pressure ^23, 24, 26^. Our results suggested that the participants had low self-efficacy to exercise and frequently encountered barriers to exercising. This corresponds to findings from other studies from USA, Nigeria and Ethiopia, who also found lower rates of physical activity among people living with diabetes; this was attributed to low self-efficacy and high perceived barriers to physical activity ^20, 21, 27, 28^.

Foot care was another self-management aspect practiced by most participants. Although foot care may not directly influence glycaemic control, it is an important self-management practice for the prevention of foot ulcers and leg amputations ^24, 29^. People living with diabetes are prone to foot ulcers due to peripheral neuropathy which result from poor glycaemic control ^23, 24^. In total, 69% of the participants in our study reported checking their feet daily and checking inside their shoes before wearing them every day. This contrasts with the findings of Assayed *et al.*, at Mangochi District Hospital in Malawi, where only 17% of diabetic patients reported inspecting their feet regularly, and 15% did not wear shoes at all ^22^. This observed difference between our study and that of Assayed *et al.* could be due to differences in settings and the quality of service delivery. Mangochi district is mostly rural and with a limited capacity of providing diabetes self-management education ^22^. Although many patients reported daily foot care, it is however not adequate considering that most of them had peripheral neuropathy. Literature shows that QECH has a high number of people living with diabetes who present late with ulcers, which may result in limb amputations ^30^.

Following a recommended healthy diet was the least regularly practiced self-management behaviour and corresponds with findings from a study that was conducted in the USA ^28^. The recommended diet for people with diabetes mainly consists of foods that have low carbohydrate, low salt, whole grains, fruits and vegetables ^23, 24, 31^ . Additionally, a healthy diet restricts fats, sweetened foods or beverages, and recommends eating of small food potions spread out evenly throughout the day ^23, 24, 31^. For their general diet, 57% of the participants reported following a healthy diet as recommended at least six days a week. The specific diet assessment showed that none of the participants were taking at least five portions of fruits and vegetables every day. This is similar to what was found in a population-based national survey conducted in Malawi, where fruit intake was on average two days per week ^25^. Following a healthy diet plan can reduce glycated haemoglobin (HbA1C) levels by up to 2% and is protective from cardiovascular and non-cardiovascular disease mortality for people living with diabetes ^23, 32^; therefore, it should be encouraged.

Self-monitoring of blood glucose was not assessed as only 12% of the participants reported to have a glucometer at home. Nevertheless, lower rates of self-monitoring of blood glucose have been reported in previous studies conducted in sub-Saharan African countries like Tanzania ^33^ and Kenya ^34^. Low rates of self-monitoring of blood glucose in people living with diabetes in Africa has been attributed to financial constraints ^33, 35^. In contrast, studies conducted in high-income countries like France ^36^, Sweden ^33^ and Italy ^35^ have reported regular self-monitoring of blood glucose among people living with diabetes. Regular monitoring of blood glucose is associated with good glycemic control ^35^.

Overall, we found that only one-third (33%) of the participants were following all (diet, exercise, foot care and medication) the recommended self-management practices. Other studies have also found that most people living with diabetes do not follow all the recommended self-management practices. A study in Ethiopia, found that only 39% were following all the self-management practices ^20^. A study in Mexico found that only 26% were following all the recommended self-care activities ^37^. In another study by Zulman *et al.* in USA, only 26% reported performing four or five of the five self-management behaviours which they assessed ^28^. Failure to follow all recommended self-management behaviours may be due to the fact that each self-management behaviour has different barriers and requires different knowledge, skills and motivation ^38^.

We found that self-efficacy was the only significant (p < 0.001) social cognitive theory factor associated with following all self-management behaviours. Many studies have also found self-efficacy as a predictor to all self-management behaviours independently or collectively ^27, 39– 43^. The social cognitive theory suggests that people with high self-efficacy set high goals for themselves, are more positive minded and have better analytical skills ^6^. Additionally, studies have shown that diabetes self-efficacy is also associated with other predictors of diabetes self-management such as health literacy, health related quality of life and social support ^43– 45^.

The other social cognitive theory constructs (outcome expectations, social support, environmental barriers and knowledge) showed no statistically significant association with satisfactory self-management. Social support and environmental barriers to self-care scores were however associated with satisfactory self-management in the univariate analysis (p < 0.05) but lost their significance in the multivariate logistic regression model. There are mixed findings on the association between outcome expectations, social support, knowledge and environmental barriers as predictors of one or more self-management behaviours. Some studies have reported an association of any of these with self-management ^27, 46– 48^ while others reported no associations ^49– 51^. Self-efficacy is, however, the main factor that regulates all the other constructs of the social cognitive theory as it influences feelings, motivation, thoughts, expectations and goals ^52^. Self-efficacy is also associated with other social cognitive constructs such as social support ^53^; therefore, more studies are required to explore further the relationship of the other social cognitive theory constructs with each other and diabetes self-management.

This study had several limitations. One of the limitations was that the participants were predominantly female. However, there were no statistically significant differences in following of self-management behaviours between males and females. Another limitation was that the study was hospital-based and recruited participants from one health facility only. Participants attending the clinic may be more compliant to self-management behaviours than those who do not come to the clinic. Additionally, generalizability of the findings is limited to central hospitals or health facilities of similar nature. Although the study identified factors associated with poor dietary behaviours, causality cannot be ascertained. Experimental studies are needed to identify locally appropriate and acceptable interventions that can improve self-efficacy in diet and all other self-management behaviours.

## Conclusion

The findings of this study show that people living with diabetes attending QECH diabetes clinic were not consistently following all the recommended self-management practices. Dietary practices were the least adhered to self-management behaviour compared to medication, foot care and exercising. Management protocols and guidelines for people living with diabetes at QECH should therefore include interventions aimed at improving self-efficacy such as exposure to role models, peer education, providing positive feedback, and counselling. We also noted that most of the people living with diabetes lacked access to resources that enabled them to perform self-monitoring of blood glucose. We recommend availability of blood glucose monitoring devices at primary health care level and even to all individuals living with diabetes to allow regular monitoring of blood glucose, which is necessary for the adjustment of medication, diet and exercise intensity.

## Data availability

### Underlying data

Figshare: Diabetes self-management and social cognitive factors. https://doi.org/10.6084/m9.figshare.9757076.v1 ^19^.

This project contains answers to each question from each respondent. The first row contains the question number from the questionnaire (see *Extended data*) to which the answer pertains.

### Extended data

Figshare: Factors associated with diabetes self-management questionnaire. https://doi.org/10.6084/m9.figshare.9757115.v1 ^7^.

Data are available under the terms of the Creative Commons Attribution 4.0 International license (CC-BY 4.0).
