# Supplementary material for: Self-management and its associated factors among people living with diabetes in Blantyre, Malawi: a cross-sectional study
Source: AAS Open Res. 2021 Dec 23;2:161. Originally published 2019 Sep 19. [Version 2] doi: 10.12688/aasopenres.12992.2 (PMC11273085; doi:10.12688/aasopenres.12992.2)
Supplement: Peer review history

# Reviewer report: Reviewer response for version 2

**DOI:** 10.21956/aasopenres.14466.r31072

**Published:** 2024-07-25

**Authors:** Elmari Deacon

**Affiliations:** 1 Faculty of Health Sciences & Department of Psychosocial Health, North-West University, Vanderbijlpark, South Africa

Thank you for the opportunity to review this manuscript. The topic of self-management in the African context is not well research and this study adds to literature.

 As for the manuscript, I would reconsider the keywords, to also include self-efficacy as this is one of the important constructs in the findings. 

 I was not sure about referring to the participants as "clients" under the method section and I have not seen the the inclusion of the sample size formula in previous manuscripts and wondered what it added to the arguments. The same goes for Figure 1 as the relationships between the different scale/constructs are not explained in text and is also not used in the discussion.

 The discussion followed well and answered the research questions. There was just a smaller typo in the last paragraph before the conclusion in the third line on p.9 "males and females. There were", should read "males and females. There were..."

Is the work clearly and accurately presented and does it cite the current literature?

No

If applicable, is the statistical analysis and its interpretation appropriate?

I cannot comment. A qualified statistician is required.

Are all the source data underlying the results available to ensure full reproducibility?

Partly

Is the study design appropriate and is the work technically sound?

Yes

Are the conclusions drawn adequately supported by the results?

Yes

Are sufficient details of methods and analysis provided to allow replication by others?

Partly

Reviewer Expertise:

Diabetes self-management

I confirm that I have read this submission and believe that I have an appropriate level of expertise to confirm that it is of an acceptable scientific standard.

# Reviewer report: Reviewer response for version 2

**DOI:** 10.21956/aasopenres.14466.r29066

**Published:** 2022-01-17

**Authors:** Nicola Brew-Sam

**Affiliations:** 1 Department of Health Services Research and Policy, Research School of Population Health, Australian National University, Canberra, Australia

Abstract:

 1." ... last seven days was 88.6% ... " - please correct in abstract without decimals (to be consistent).

Introduction:

 2. "These key concepts are the factors that influence human action, motivation and wellbeing and hence are hypothesized to be associated with diabetes self-management for this present study ^8^. Self-management was assesed using the Summary of Diabetes Self-care Activities (SDSCA) measure (Toobert). The aim for this study was to assess the level of self-management and identify factors associated with practicing all self-management behaviours among adults living with diabetes at QECH. This is part of a larger study exploring self-management practices and experiences among people living with diabetes attending the QECH diabetes clinic."

 I would change the paragraph as follows for better readability and clarity:

 These key concepts are the factors that influence human action, motivation and wellbeing ^8^ [remove second part of sentence, aim has to be mentioned before hypothesis].

 [new paragraph] The aim for this study was to assess the level of self-management and identify SCT factors associated with practicing all self-management behaviours among adults living with diabetes at QECH. This is part of a larger study exploring self-management practices and experiences among people living with diabetes attending the QECH diabetes clinic.

 The sentence "Self-management was assesed using the Summary of Diabetes Self-care Activities (SDSCA) measure (Toobert)" should be moved to methods, and contains a spelling mistake "assesed".

Methods:

 3. "standard face-to-face surveys" - standardized?

 4. "conducted to collect demographics, clinical, diabetes knowledge, self-efficacy, outcome expectations, social support, environmental barriers and diabetes self-management data" - which ones are SCT factors? Please clarify already here, e.g.,: to collect demographics, clinical history, diabetes self-management data, and the SCT factors diabetes knowledge, self-efficacy, outcome-expectations, environmental barriers to proper self-management and social support. Moreover, "history" is missing for "clinical".

Inclusion criteria:

 5. " Recruitment of clients was done " - better undertaken.

 6. Remove the "where" in formula explanation.

 7. "at the 95% confidence level and at 4% level of precision" - confidence interval?

Instruments:

 8."with Cronbach’s alpha coefficient α" - the α can be removed or replace the "alpha" (duplicate).

 9. Remove space in "0.74 to 0. 93".

 10. "environmental barriers of the participants" - better: environmental barriers as perceived by the participants.

 11. Why was Cronbach's alpha not reported for environmental barriers (DAT) but all other scales? Likert scales here too? This is a bit unclear, please add scale information and alpha if applicable.

Data analysis:

 12. "who were therefore excluded in the final analysis" - better: from the final analysis.

Results:

 13. Why is there such a discrepancy of participation regarding gender (much more women)?

 14. Table 2: Spell out FBG and BP, not all readers are familiar with these abbreviations; duration of what? Add "duration of living with diabetes" or similar.

 Participant questionnaire responses:

 15. "The median knowledge score on the diabetes knowledge questionnaire was 14 (IQR 12–16) with lowest knowledge scores being on causes of diabetes, importance of diet and exercising and recognition of hypoglycemia or hyperglycemia. The median self-efficacy score was 8.6 (IQR 7.5–9.5), and the participants had lower self-efficacy on eating evenly spaced meals regularly and exercising for at least 30 minutes three..."

 Why were median scores used and not mean scores?

 From my understanding you would have to add scale information here to set the scores in relation to the various scales? The scores are only interpretable if the reader knows if it was e.g., a 5-7-10 point likert scale here, right?

 Univariate and multivariate analysis:

 16. "To investigate the factors associated with adhering to all the self-management behaviours, univariate and multivariate regression analyses were done." - better: calculated.

 17. The most important section on uni- and multi-variate analysis is very short, and could be extended.

Discussion:

 18."Our results suggested that the participants had low self-efficacy to exercise and frequently encountered barriers to exercising." - I am missing more detailed findings on this conclusion in the results section. The results should be more detailed. You just report satisfactory self-management as the main outcome variable in the multivariate analysis, but did you find any associations of SCT factors when looking at the single self-management practices as reported here (exercise)?

 19. How does the absence of BG monitoring with a glucometer affect diabetes health outcomes? Are there any studies that could be cited here? Monitoring is a very crucial aspect, and it is a major factor that could impact outcomes here.

Overall:

 20. The number of decimals should be consistent throughout; the use of present/past should be consistent in the discussion.

Is the work clearly and accurately presented and does it cite the current literature?

Yes

If applicable, is the statistical analysis and its interpretation appropriate?

Partly

Are all the source data underlying the results available to ensure full reproducibility?

Yes

Is the study design appropriate and is the work technically sound?

Yes

Are the conclusions drawn adequately supported by the results?

Yes

Are sufficient details of methods and analysis provided to allow replication by others?

Partly

Reviewer Expertise:

Health service research, diabetes research, mHealth/eHealth

I confirm that I have read this submission and believe that I have an appropriate level of expertise to confirm that it is of an acceptable scientific standard, however I have significant reservations, as outlined above.

# Reviewer report: Reviewer response for version 1

**DOI:** 10.21956/aasopenres.14076.r28577

**Published:** 2021-05-24

**Authors:** Nicola Brew-Sam

**Affiliations:** 1 Department of Health Services Research and Policy, Research School of Population Health, Australian National University, Canberra, Australia

Thank you very much for the interesting study, I think it is well conducted with some minor weaknesses in the manuscript. Please find my suggestions below:

  

1. The gender distribution could be added to the abstract.
2. n=494 in the abstract's result section only applies for one result, and the other results refer to N=510? This is a bit unclear. I would suggest removing n=494 from the abstract.
3. Percentages should be reported consistently without decimals 88.6% = 89%
4. "Although the study by Cohen *et al. *had shown that people living with diabetes in Malawi had poorly controlled glucose, little was known about their self-management behaviours especially regarding diet, exercising, self-monitoring of blood glucose, medication adherence, and foot care" - this is a repetition of the first paragraph.
5. "A systematic review of randomized controlled trials of SCT-based inter- ventions showed effectiveness in improving diet and exercise behaviour in cancer survivors" - I would suggest to report another example on diabetes here, the cancer example doesn't fully fit. There is plenty of diabetes research focusing on SCT.
6. "A cross-sectional design" - please specify which data collection methods were used (standardised face-to-face survey?).
7. "95% confidence **level**" - better: confidence interval.
8. "The proportion was estimated to be within 0.04 (4%), using the 95% confidence level a sample of 462 patients living with diabetes would be required" - I don't understand this, please explain.
9. "Interviewer-administered questionnaire" - is that a standardized face-to-face survey? Or semi-structured interviews?
10. Page 4 Toobert, not "Toolbert".
11. "Measure has been **proved** from previous studies" - proven.
12. "For all the questions, a higher number of days indicated satisfactory self-management" - what is a higher number? Which scale values were rated as satisfactory? Please explain this, as it is relevant for the results.
13. "The tool had **showed **construct validity" - had shown.
14. "Cronbach’s coefficient alpha" - better: Cronbach’s alpha coefficient.
15. "For **doing** a particular behaviour" - better: executing.
16. "Univariate logistic regression was **done** to investigate" - better: used.
17. "The unadjusted logistic regression analyses showed that satisfactory self-management was associ- ated with self-efficacy, social support, outcome expectations and diabetes barrier score (the results are shown in Table 3)." - outcome expectations is not significant in Table 3, why is it still included in these results? Please explain.
18. Include a sub-heading for "study limitations".
19. Check paper again for spelling and grammar, there are some mistakes as outlined above.

Is the work clearly and accurately presented and does it cite the current literature?

Yes

If applicable, is the statistical analysis and its interpretation appropriate?

Partly

Are all the source data underlying the results available to ensure full reproducibility?

Yes

Is the study design appropriate and is the work technically sound?

Yes

Are the conclusions drawn adequately supported by the results?

Yes

Are sufficient details of methods and analysis provided to allow replication by others?

Partly

Reviewer Expertise:

Health service research, diabetes research, mHealth/eHealth

I confirm that I have read this submission and believe that I have an appropriate level of expertise to confirm that it is of an acceptable scientific standard, however I have significant reservations, as outlined above.

# Response

**Published:** 2021-12-10

**Authors:** Chimwemwe Kwanjo Banda

**Affiliations:** University of Malawi College of Medicine, Malawi

Approved With Reservations

 Thank you very much for the interesting study, I think it is well conducted with some minor weaknesses in the manuscript. Please find my suggestions below:

 The gender distribution could be added to the abstract.

 The gender distribution has been added in the abstract

 n=494 in the abstract's result section only applies for one result, and the other results refer to N=510? This is a bit unclear. I would suggest removing n=494 from the abstract.

 N = 494 has been removed. This number was used because 16 people with diabetes who were recruited in the study were not on diabetes medication.

 Percentages should be reported consistently without decimals 88.6% = 89%

 Thank you. All percentages have been rounded up to whole numbers and decimals removed

 "Although the study by Cohen et al. had shown that people living with diabetes in Malawi had poorly controlled glucose, little was known about their self-management behaviours especially regarding diet, exercising, self-monitoring of blood glucose, medication adherence, and foot care" - this is a repetition of the first paragraph.

 The sentence in the previous paragraph has been deleted.

 "A systematic review of randomized controlled trials of SCT-based inter- ventions showed effectiveness in improving diet and exercise behaviour in cancer survivors" - I would suggest to report another example on diabetes here, the cancer example doesn't fully fit. There is plenty of diabetes research focusing on SCT.

 The cancer article has been removed and replaced with one reporting SCT in diabetes.

 "A cross-sectional design" - please specify which data collection methods were used (standardised face-to-face survey?).

 The data collection methods have been specified as follows: “A cross-sectional design using standardised face-to-face survey was conducted” 

 "95% confidence level" - better: confidence interval.

 Thank you. Confidence level has been changed to confidence interval

 "The proportion was estimated to be within 0.04 (4%), using the 95% confidence level a sample of 462 patients living with diabetes would be required" - I don't understand this, please explain.

 This has been rephrased to add clarity:

 To determine the true proportion with satisfactory self-management at the 95% confidence level and at 4% level of precision, a minimum sample size of 462 people living with diabetes was required *.*

 The formula used to calculate the sample size has been given.

 "Interviewer-administered questionnaire" - is that a standardized face-to-face survey? Or semi-structured interviews?

 Yes- it is standardized face-to-face survey. “Interviewer administered questionnaire” has been replaced with “standardized face-to-face survey for clarity.

 Page 4 Toobert, not "Toolbert".

 The name has been corrected from Toolbert to Toobert.

 "Measure has been proved from previous studies" - proven.

 This has been corrected

 "For all the questions, a higher number of days indicated satisfactory self-management" - what is a higher number? Which scale values were rated as satisfactory? Please explain this, as it is relevant for the results

 Thank you. This has been explained as follows: “Self-management was considered satisfactory if a person reported following the recommended practices related to diet, medication, and foot care on all days in the past seven days, and being active for at least 30 minutes on three days in the past seven day.

 "The tool had showed construct validity" - had shown.

 This has been corrected

 "Cronbach’s coefficient alpha" - better: Cronbach’s alpha coefficient.

 This has been corrected

 "For doing a particular behaviour" - better: executing.

 “Executed” used instead of “doing”

 "Univariate logistic regression was done to investigate" - better: used.

 “done” replaced with “used”

 "The unadjusted logistic regression analyses showed that satisfactory self-management was associ- ated with self-efficacy, social support, outcome expectations and diabetes barrier score (the results are shown in Table 3)." - outcome expectations is not significant in Table 3, why is it still included in these results? Please explain.

 Thank you for noting this. Outcome expectations were not associated with self-management as shown in Table 3. This has been corrected in the text

 Include a sub-heading for "study limitations".

 This has been included

 Check paper again for spelling and grammar, there are some mistakes as outlined above.

 Thank you. This has been done.

  

- Is the work clearly and accurately presented and does it cite the current literature?

Yes

- Is the study design appropriate and is the work technically sound?

Yes

- Are sufficient details of methods and analysis provided to allow replication by others?

Partly

- If applicable, is the statistical analysis and its interpretation appropriate?

Partly

- Are all the source data underlying the results available to ensure full reproducibility?

Yes

- Are the conclusions drawn adequately supported by the results?

Yes

 Thank you.

 More details have been added as described above.

 This has been improved as suggested above.

 Thank you.

# Reviewer report: Reviewer response for version 1

**DOI:** 10.21956/aasopenres.14076.r27803

**Published:** 2020-08-26

**Authors:** Fazlyn Petersen

**Affiliations:** 1 Department of Information Systems, University of the Western Cape, Cape Town, South Africa

**Is the work clearly and accurately presented and does it cite the current literature? **Partly:

1. “The inpatient mortality for the people admitted due to diabetes at QECH is **about **19%” - can the actual value not be presented instead?
2. “A previous survey at QECH by Cohen *et al.*, conducted **10 years** before the present study” - more recent studies should be used. 
3. Why is SCT for cancer being used when there is available literature for SCT and diabetes?
4. There is more recent literature available on diabetes self-management and SCT such as Ghoreishi *et al.* (2019 ^1^), Bourne *et al.* (2020 ^2^), Thojampa and Sarnkhaowkhom (2019 ^3^) and Mirzaei-Alavijeh and Jalilian (2019 ^4^).
5. The link between SCT and diabetes self-management could be better explained by using the references above, among others. 

**Is the study design appropriate and does the work have academic merit?**

1. The work does have merit but I feel that SDSCA should be introduced in the last paragraph of the introduction. "Some key concepts of the social cognitive theory are self-efficacy, health knowledge, health goals, outcome expectations, and environmental impediments and facilitators". The use of SDSCA is not explicit in this sentence. 

**Are sufficient details of methods and analysis provided to allow replication by others?**

1. Inclusion and exclusion criteria are well defined.
2. Sample size considerations - why is a 10-year-old study used as a basis instead of a sample size calculator like Raosoft? http://www.raosoft.com/samplesize.html
3. It would be clearer to see a research model diagrammatically presented.

**If applicable, is the statistical analysis and its interpretation appropriate?**

1. This tool had been used in previous studies with a Cronbach’s coefficient α of 0.85 and a test-retest validity of 0.8". As this survey is a combination of other surveys with adaptations made, I would recommend that Construct Reliability and Validity and Discriminant Validity be tested for the final survey used. At least Cronbach alpha should have been tested in this study too.
2. I find Table 3 rather difficult to understand. Why are there p-values shown for demographic variables? I assume that these are for testing the associations. 
3. I use structured equation modelling to test research models and hypotheses so this method is not very clear to me. "…social cognitive theory constructs (outcome expectations, social support, environmental barriers and knowledge) showed no statistically significant association with satisfactory self-management." - where are these hypotheses represented? I was expecting to see an independent and dependent variable in Table 3 to better understand the p-values.

- It mentions "causality cannot be ascertained" but this needs to be clarified.
- **Limitations** - "However, there were no statistically significant differences in following of self-management behaviours between males and females" - this statement is missing a reference.

**Are all the source data underlying the results available to ensure full reproducibility?** Yes, the survey and results are available.

**Are the conclusions drawn adequately supported by the results?** Yes.

Is the work clearly and accurately presented and does it cite the current literature?

Partly

If applicable, is the statistical analysis and its interpretation appropriate?

Partly

Are all the source data underlying the results available to ensure full reproducibility?

Yes

Is the study design appropriate and is the work technically sound?

Partly

Are the conclusions drawn adequately supported by the results?

Yes

Are sufficient details of methods and analysis provided to allow replication by others?

Partly

Reviewer Expertise:

The use of technology to improve diabetes self-management.

I confirm that I have read this submission and believe that I have an appropriate level of expertise to confirm that it is of an acceptable scientific standard, however I have significant reservations, as outlined above.

## References

[1] : Self-care behaviors in patients with type 2 diabetes: Education intervention base on social cognitive theory. *Diabetes Metab Syndr* .13(3) : 10.1016/j.dsx.2019.04.045 2049-2056 10.1016/j.dsx.2019.04.045 31235135

[2] : Mapping behavior change techniques to characterize a social cognitive theory informed physical activity intervention for adults at risk of type 2 diabetes mellitus. *Transl Behav Med* .2020;10(3) : 10.1093/tbm/ibz008 705-715 10.1093/tbm/ibz008 30715534

[3] : The Social Cognitive Theory with Diabetes: Discussion. *International Journal of Caring Sciences* .2019;12(2) :1251 Reference source

[4] : A psychometric analysis of the socio-cognitive determinants questionnaire of type 2 diabetes prevention among a group at risk. *Iranian Journal of Endocrinology and Metabolism* .2019;20(5) :224-234 Reference source

# Response

**Published:** 2021-12-10

**Authors:** Chimwemwe Kwanjo Banda

**Affiliations:** University of Malawi College of Medicine, Malawi

**Is the work clearly and accurately presented and does it cite the current literature? **Partly:

1. “The inpatient mortality for the people admitted due to diabetes at QECH is **about **19%” - can the actual value not be presented instead?

The sentence has been rephrased and reads: The inpatient mortality for the people admitted due to diabetes at QECH is 19%.

  

1. “A previous survey at QECH by Cohen *et al.*, conducted **10 years** before the present study” - more recent studies should be used. 

Noted. However there was no available literature on recent studies looking at self-management or glycemic control at QECH other than the Cohen *et al*. study.  This has been highlighted in the paper

  

1. Why is SCT for cancer being used when there is available literature for SCT and diabetes?

The reference has been replaced with one focusing on diabetes mellitus and STC.

  

1. There is more recent literature available on diabetes self-management and SCT such as Ghoreishi *et al.* (2019 ^1^ ), Bourne *et al.* (2020 ^2^ ), Thojampa and Sarnkhaowkhom (2019 ^3^ ) and Mirzaei-Alavijeh and Jalilian (2019 ^4^

Thank for the suggestions. These references have been included.

  

1. The link between SCT and diabetes self-management could be better explained by using the references above, among others. 

Noted. This has been done

**Is the study design appropriate and does the work have academic merit?**

  

1. The work does have merit but I feel that SDSCA should be introduced in the last paragraph of the introduction. "Some key concepts of the social cognitive theory are self-efficacy, health knowledge, health goals, outcome expectations, and environmental impediments and facilitators". The use of SDSCA is not explicit in this sentence. 

Thank you. A sentence has been added in the introduction paragraph to introduce the SDSCA.

**Are sufficient details of methods and analysis provided to allow replication by others?**

  

1. Inclusion and exclusion criteria are well defined.

Thank you.

  

1. Sample size considerations - why is a 10-year-old study used as a basis instead of a sample size calculator like Raosoft? http://www.raosoft.com/samplesize.html

Noted. We will consider using the given site in future. We used the old study because that was the only available local study to help us determine the proportion estimated to have good self-management at the time of the study.

  

1. It would be clearer to see a research model diagrammatically presented.

A research model has been included.

**If applicable, is the statistical analysis and its interpretation appropriate?**

  

1. This tool had been used in previous studies with a Cronbach’s coefficient α of 0.85 and a test-retest validity of 0.8". As this survey is a combination of other surveys with adaptations made, I would recommend that Construct Reliability and Validity and Discriminant Validity be tested for the final survey used. At least Cronbach alpha should have been tested in this study too.

Thank you for that recommendation. Cronbach alpha for this study has been calculated and included in the paper.

  

1. I find Table 3 rather difficult to understand. Why are there p-values shown for demographic variables? I assume that these are for testing the associations. 

Table 3 has been simplified to include the association of self-management and social cognitive constructs (knowledge, self-efficacy, outcome expectations, social support and barriers) only . P values are used because the table shows results of univariate logistic regression analysis that tested independent associations between predictor variables (demographic characteristics, clinical factors and social cognitive constructs).

  

1. I use structured equation modelling to test research models and hypotheses so this method is not very clear to me. "…social cognitive theory constructs (outcome expectations, social support, environmental barriers and knowledge) showed no statistically significant association with satisfactory self-management." - where are these hypotheses represented? I was expecting to see an independent and dependent variable in Table 3 to better understand the p-values.

We used logistic regression analysis. The dependent variable was self-management dichotomized as satisfactory or unsatisfactory. Based on the conceptual framework guiding the study, our hypothesis was that the social cognitive theory constructs are associated with self-management. We had three categories of independent variables (1-demographic characteristics, 2- clinical characteristics and 3- social cognitive theory variables). The first step of the analysis was to test the independent associations between each of the independent variables and the dependent variable. Table 3 shows the results of the univariate logistic regression analysis, which shows the association between each of the dependent variable with self-management.

 The second step was the multivariate regression analysis where independent variables that showed association with the dependent variable (self-management) were included.

  

1. It mentions "causality cannot be ascertained" but this needs to be clarified.

The sentence has been rephrased as follows: “Since this was a cross-sectional study, we were only able to identify factors associated with diabetes self-management and not the causes”

  

1. **Limitations** - "However, there were no statistically significant differences in following of self-management behaviours between males and females" - this statement is missing a reference.

This sentence has been rephrased as follows: However, at univariate analysis, we found no statistically significant differences in following of self-management behaviours between males and females.

**Are all the source data underlying the results available to ensure full reproducibility?** Yes, the survey and results are available.

 Thank you

**Are the conclusions drawn adequately supported by the results?** Yes.

 Thank you

  

- Is the work clearly and accurately presented and does it cite the current literature?

Partly

 Thank you. This has been improved and literature updated.

- Is the study design appropriate and is the work technically sound?

Partly

 This has been improved

- Are sufficient details of methods and analysis provided to allow replication by others?

Partly

 This has been improved.

- If applicable, is the statistical analysis and its interpretation appropriate?

Partly

 This has been improved and more details of the analysis are given.

- Are all the source data underlying the results available to ensure full reproducibility?

Yes

- Are the conclusions drawn adequately supported by the results?

Yes

 Thank you.
